# Relational Sanctity as a Contributor to Forgiveness: Dyadic Data From Older African American Couples

**DOI:** 10.1093/geroni/igab046.3338

**Published:** 2021-12-17

**Authors:** Antonius Skipper, Andrew Rose, Alex Reeves, Jhazzmyn Joiner, Ethan Jones

**Affiliations:** 1 Georgia State University, Atlanta, Georgia, United States; 2 Texas Tech University, Lubbock, Texas, United States

## Abstract

Although research finds that healthy romantic relationships can provide several benefits in older adulthood, few studies examine the relational characteristics of older African American couples. Further, despite positive associations between religiosity and age, particularly among African Americans, a dearth of dyadic data consider the importance of religious constructs within the relationships of older African Americans. To address this gap, this study utilized dyadic data from the Strong African American Couples Project to examine the interconnection between relational sanctity and forgiveness among married and cohabiting older African American couples. A total of 194 African American couples (146 married and 48 cohabiting) aged 50 to 86 years were included in the analysis, and Actor Partner Independence Models were used to test the relational effects of sanctity and forgiveness. Findings revealed that no significant effects existed when women’s relational sanctity was the predictor variable. However, men’s relational sanctity had a significant positive association with both his own forgiveness of his partner and his perception of his partner’s forgiveness. These findings have valuable implications for professionals engaging older African American couples. First, this study helps to counter the often deficit-focused literature on African American couples by highlighting the potentially stabilizing influence of viewing one’s relationship as sacred. Second, this study offers a rare glimpse into the aspects of men’s religiosity that may be more consequential than women’s. Both practitioners and clergy could use this information to inform counseling efforts that seek to build on the strengths of married and cohabiting older African American couples.

